# Minimal clinically important difference and minimal detectable change of the World Health Organization Disability Assessment Schedule 2.0 (WHODAS 2.0) amongst patients with chronic musculoskeletal pain

**DOI:** 10.1177/0269215520942573

**Published:** 2020-07-27

**Authors:** Niina Katajapuu, Ari Heinonen, Mikhail Saltychev

**Affiliations:** 1 Faculty of Sports and Health Sciences, University of Jyväskylä, Jyväskylä, Finland; 2 Faculty of Health and Wellbeing, Turku University of Applied Sciences, Turku, Finland; 3 Department of Physical and Rehabilitation Medicine, Turku University Hospital and University of Turku, Turku, Finland

**Keywords:** Whodas, minimal clinically important difference, minimal detectable change, musculoskeletal pain

## Abstract

**Objectives:**

The aim of this study is to estimate a minimal clinically important difference (MCID) and a minimal detectable change (MDC) of the 12-item WHODAS 2.0 amongst patients with chronic musculoskeletal pain.

**Design:**

Cross-sectional cohort study.

**Setting:**

Outpatient Physical and Rehabilitation Medicine clinic.

**Subjects:**

A total of 1988 consecutive patients with musculoskeletal pain.

**Interventions:**

A distribution-based approach was employed to estimate a minimal clinically important difference, a minimal detectable change, and a minimal detectable percent change (MDC%).

**Results:**

The mean age of the patients was 48 years, and 65% were women. The average intensity of pain was 6,3 (2.0) points (0–10 numeric rating scale) and the mean WHODAS 2.0 total score was 13 (9) points out of 48. The minimal clinically important difference ranged between 3.1 and 4.7 points. The minimal detectable change was 8.6 points and minimal detectable % change was unacceptably high 66%.

**Conclusions:**

Amongst patients with chronic musculoskeletal pain, the 12-item WHODAS 2.0 demonstrated a high minimal detectable change of almost nine points. As the minimal detectable change exceeded the level of minimal clinically important difference, nine points were considered to be the amount of change perceived by a respondent as clinically significant.

## Introduction

The World Health Organization Disability Assess-ment Schedule 2.0 (WHODAS 2.0) is a generic tool to assess functioning in diverse situations.^1–3^ While the WHODAS 2.0 has widely been used in clinical practice and research, the interpretation of results, obtained from the WHODAS 2.0 responses, have not been well defined.^4,5^

The interpretation of test results relies heavily on such characteristics as minimal clinically important difference (“MCID”) and minimal detectable change (“MDC”). While changes in test score may be statistically significant, they are not necessarily perceived by patients as clinically significant. This is especially true when the results are obtained from a large sample – a very small difference may become statistically significant while its practical importance perceived by patients is negligible. Minimal clinically important difference describes the smallest amount of change or difference that might be considered important by patients or clinicians.^6^ There are two common method to calculate a minimal clinically important difference: an anchor-based and a distribution-based. There is no general agreement on which method is preferable. Probably, they both have their pros and cons in different particular situations. The anchor can be either an objective or subjective measure (e.g. question about mild improvement noticed by a patient or a clinician). An anchor-based method reflects the patient’s of clinician’s point of view. In turn, a distribution-based method is based explicitly on the statistical variability of obtained scores. A minimal detectable change is the smallest amount of change or difference that is not the result of measurement error.^7^ In an clinically ideal world, the minimal clinically important difference must exceed the level of minimal detectable change. If minimal clinically important difference is less than minimal detectable change, then the observed result below the level of minimal detectable change may be caused by chance and not by the true difference in scores even if the result exceeds the level of minimal clinically important difference.^8^

Thus, without knowledge on minimal clinically important difference and minimal detectable change, the clinical meaning of the WHODAS 2.0 total score estimates remains unclear. The minimal clinically important difference of the 12-item WHODAS 2.0 has been established by a single study amongst patients with anxiety and stress disorders.^9^ In that study, an anchor-based method has been used, and the minimal clinically important difference has been estimated around three points for a less strict model and six to seven points for a stricter model. The minimal detectable change of the WHODAS 2.0 has also recently been reported by a single study amongst institutionalized ambulatory older adults as 10 points.^10^ So far, there have not been reports on the minimal clinically important difference or minimal detectable change of the WHODAS 2.0 amongst patients with musculoskeletal health conditions. Due to the WHODAS 2.0 score’s multidimensionality and, thus, potentially high level of minimal detectable change, the trustworthiness of the WHODAS 2.0 total score has been questioned.^2,11^

The objective of this study was to estimate the minimal clinically important difference and minimal detectable change of 12-item WHODAS 2.0 amongst people with chronic musculoskeletal pain.

## Methods

Data for this study were derived from the Turku ICF Study (T54/2012) approved by the ethics committee of Hospital District of Southwest Finland (ETMK 60/180/2012). Participants provided their written informed consent for participation. This was a cross-sectional study amongst 3150 consecutive patients who were seen in an outpatient Physical and Rehabilitation Medicine clinic of university hospital between April 2014 and February 2017. The survey was sent to the patients and filled up before a physician appointment. The survey included 12-item WHODAS 2.0 questionnaire and questions on demographics, pain intensity, and perceived general health.

### Self-administered 12-item WHODAS 2.0

The self-administered 12-item WHODAS 2.0 contains 12 items covering the most common limitations of functioning appearing in general population (Appendix A). The questionnaire covers limitations during the last 30 days. A Likert-like scale is used to define the severity of limitation with 0 denoting “no limitation” and 4 denoting “extreme limitation or inability to function.” The total score is the sum of all 12 items where a score of 48 points represents the worst possible restriction.^1^

### Independent variables

*Age* was defined in full years at the time of visiting the clinic. *Pain intensity* was assessed using a 11-point numeric rating scale with 0 denoting “no pain” and 10 denoting “worst possible pain.” *Educational level* was dichotomized “further education” (equivalent “further education or higher” in UK) versus “no further education” (equivalent of “primary and secondary education” in UK). *Body mass index* was calculated as a body mass divided by a squared body height (kg/m^2^). *Perceived general health* status was assessed on a 4-point scale where 0 indicated best possible and 3 worst possible health. Main diagnoses were defined using the International Classification of Diseases, 10th edition

### Statistical analysis

The results were reported as means, standard deviations, and standard errors, medians, ranges, and interquartile ranges when appropriate. The internal consistency was assessed by Cronbach’s alpha considering α ⩾ 0.9 excellent, 0.8 ⩽ α < 0.9 good, 0.7 ⩽ α < 0.8 acceptable, 0.6 ⩽ α < 0.7 questionable, 0.5 ⩽ α < 0.6 poor, and α < 0.5 unacceptable.

To describe the variability between an individual’s observed score and the true score, standard error of measurement (SEM) was calculated as SEM = SD × √(1 – r_xx_) where r_xx_ is reliability coefficient of the test – in this case, Cronbach’s alpha.^12^ Since the data were cross-sectional and no patients’ opinion on perceived change in functioning was available as an anchor, a distribution-based approach was employed to estimate minimal clinically important difference for the WHODAS 2.0. Three different formulas were used for the task:^13–18^

1) Minimal clinically important difference = standard error of measurement
2) Minimal clinically important difference = 0.5 × standard deviation
3) Minimal clinically important difference = 0.33 × standard deviation

The minimal detectable change was calculated as 1.96 × standard error of measurement × √2. The minimal detectable change was also expressed as a percentage (“MDC%”) – an estimate that is independent of the units of measurement. Representing the relative amount of random measurement error, the minimal detectable % change was calculated as (minimal detectable change /observed mean WHODAS total score) × 100. The minimal detectable % change <30% was considered acceptable and <10% excellent^19,20^

All the analyses were conducted using Stata/IC Statistical Software: Release 15. College Station (StataCorp LP, TX, USA).

## Results

Of 3150 patients visiting the clinic, 1988 (63%) participated the study. The patients were 47.6 (6.3) years old and 1297 (65%) were women. The average intensity of pain was 6.3 (2.0) points on a numeric rating scale. The general health median was 1 (range 0 to 4, IQR 1 to 2) (Table 1). The majority of the patients were referred to the clinic due to non-specific chronic pain in their low back, neck, extremities, or soft tissue in general. Due to the national guidelines, patients with rheumatoid arthritis, severe osteoarthritis, or fractures were referred to other specialized clinics. Thus, only one patient had a main diagnosis of rheumatoid arthritis, 0.3% had diagnoses of traumas, and 2% had diagnoses of primary osteoarthritis. Most of the patients (*n* = 1746, 88%) had a main diagnosis “M” – “Diseases of the musculoskeletal system and connective tissue.” The most frequent single diagnoses were “M54 Dorsalgia” (*n* = 781, 39%) and “M79 Other soft tissue disorders” (*n* = 202, 10%). The patients’ characteristics are presented in Table 1.

**Table 1.** Demographic characteristics and the WHODAS 2.0 total score.

Variable	Total
Age (mean and standard deviation), years	47.6 (6.3)
Body mass index (mean and standard deviation), kg/cm^2^	27.4 (5.7)
Pain (mean and standard deviation), points	6.3 (2.0)
Educational level (absolute proportions and percentage)	
No further education	1258 (67%)
Further education or higher	609 (33%)
WHODAS 2.0 (mean and standard deviation), points	13.1 (9.4)
WHODAS 2.0 (median, range, and interquartile range [IQR]), points	12 (0 to 48, IQR 6 to 19)

WHODAS: World health Organization Disability Assessment Schedule.

**Figure 1.**
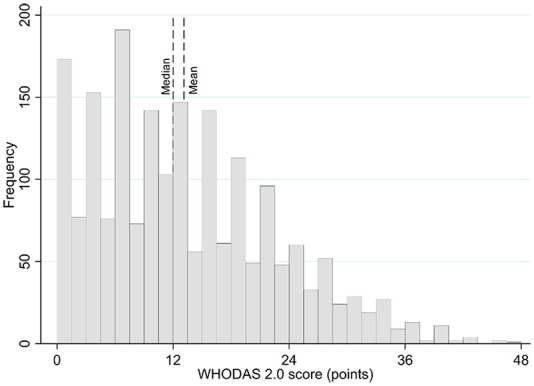
Histogram of the WHODAS 2.0 total score distribution. WHODAS: World Health Organization Disability Assessment Schedule.

The distribution of WHODAS 2.0 total score was abnormal with shift towards mild disability levels (Figure 1). However, the median and mean estimates were alike and thus, the distribution was considered close to normal enough to proceed with calculations based on mean and standard deviation. The Cronbach’s alpha was good 0.89. The mean WHODAS 2.0 total score was 13.1 (9.4) and median 12 (Inter quartile range 6–19, range 0–48) points. Based on three different calculation formulas, the minimal clinically important difference estimates for the WHODAS 2.0 were 3.10 (calculated as standard error of measurement), 3.09 (calculated as one third of standard deviation), to 4.68 (calculated as half of standard deviation) points. The minimal detectable change was 8.6 points exceeding the level of minimal clinically important difference and minimal detectable percentage change was unacceptably high 66%.

## Discussion

Amongst almost 2000 patients with chronic musculoskeletal pain, the 12-item WHODAS 2.0 total score demonstrated a minimal clinically important difference of three to five points with minimal detectable change of up to 9 points exceeding a minimal clinically important difference almost twice. The minimal detectable % change showed that almost 70% change in WHODAS 2.0 total score should be expected before patients or clinicians might detect the change clinically.

While the large study sample advocates for the trustworthiness of the findings, the generalization of the results may be compromised by the study’s cross-sectional design. Indeed, there were not longitudinal data to re-check the estimates by using an anchor-based approach with patients’ real responses on the changed clinical situation. It has to be kept in mind that minimal clinically important difference and minimal detectable change are always statistical approximations, which could be different in real-world circumstances. The WHODAS 2.0 scores were distributed abnormally in the studied sample with most of the patients perceived only mild limitations of functioning. Therefore, caution is needed when generalizing the results amongst populations with more severe limitations.

Consistent with the results of this study, a study by Silva et al. has recently set the minimal detectable change of 12-item WHODAS in institutionalized elderly to 9.6 points.^10^ Respectively, the size of minimal clinically important difference seen in the present study was similar to the estimates reported previously by a study amongst patients with anxiety and stress disorders.^9^ The results indirectly support previous reports on the potential unreliability of WHODAS 2.0 total score due to multidimensionality and a significant floor effect. A recent review suggested that 12-item WHODAS 2.0 is a multidimensional scale and it might be more useful when used to create a functioning profile than when providing a single total score.^2^ A substantial floor effect of the 12-item WHODAS 2.0 has been seen in two studies.^21,22^ All these previous findings may explain the high estimates of minimal clinically important difference and minimal detectable change seen in the present study.

Further research in different populations is recommended. To confirm the results by using an anchor-based approach, longitudinal design is needed. The WHODAS 2.0 can be scored using a simple addition of individual items’ scores (used in this study) or a more complex scheme taking into account the weights of different domains included into the WHODAS 2.0. Using that second scheme might affect the observed estimates and this possibility could be investigated by further research.

**Clinical messages**

- Amongst patients with chronic musculoskeletal pain, the 12-item WHODAS 2.0 demonstrated a high minimal detectable change of almost nine points.
- As the minimal detectable change exceeded the level of minimal clinically important difference, nine points were considered to be the amount of change perceived by a respondent as being clinically significant.

## Appendix A. The 12-item WHODAS 2.0

In the past 30 days, how much difficulty did you have in:

1. Standing for long periods such as 30 minutes?
2. Taking care of your household responsibilities?
3. Learning a new task, for example, learning how to get to a new place?
4. How much of a problem did you have in joining in community activities (e.g. festivities, religious or other activities) in the same way as anyone else can?
5. How much have you been emotionally affected by your health problems?
6. Concentrating on doing something for 10 minutes?
7. Walking a long distance such as a kilometer (or equivalent)?
8. Washing your whole body?
9. Getting dressed?
10. Dealing with people you do not know?
11. Maintaining a friendship?
12. Your day-to-day work?

(0) None; (1) Mild; (2) Moderate; (3) Severe; (4) Extreme/Cannot do
